# Trajectories of adolescent life satisfaction

**DOI:** 10.1098/rsos.211808

**Published:** 2022-08-03

**Authors:** Amy Orben, Richard E. Lucas, Delia Fuhrmann, Rogier A. Kievit

**Affiliations:** ^1^ MRC Cognition and Brain Sciences Unit, University of Cambridge, Cambridge, UK; ^2^ Department of Psychology, Michigan State University, East Lansing, MI, USA; ^3^ Department of Psychology, King's College London, London, UK; ^4^ Donders Institute for Brain, Cognition and Behaviour, Radboud University Medical Center, Nijmegen, Gelderland, The Netherlands

**Keywords:** life satisfaction, latent growth models, lifespan development, adolescents, sex differences, subjective wellbeing

## Abstract

Increasing global policy interest in measuring and improving population wellbeing has prompted academic investigations into the dynamics of lifespan life satisfaction. Yet little research has assessed the complete adolescent age range, although it harbours developmental changes that could affect wellbeing far into adulthood. This study investigates how life satisfaction develops throughout the whole of adolescence, and compares this development to that in adulthood, by applying exploratory and confirmatory latent growth curve modelling to UK and German data, respectively (37 076 participants, 10–24 years). We find a near universal decrease in life satisfaction during adolescence. This decrease is steeper than at any other point across adulthood. Further, our findings suggest that adolescent girls' life satisfaction is lower than boys’, but that this difference does not extend into adulthood. The study highlights the importance of studying adolescent subjective wellbeing trajectories to inform research, policy and practice.

## 1. Introduction

The past decade has seen global wellbeing become progressively enumerated, tracked and analysed in a bid to understand and maximize human happiness [1]. Efforts range from the regular publication of wellbeing reports (e.g. [2]) to calls for wellbeing indices to replace the predominant economic and political metrics of gross domestic product (GDP) and gross national product in steering national and international politics [3,4]. Responding to these developments, psychologists and other behavioural scientists have been increasingly attending to wellbeing as a valuable area of academic research [5,6].

The resulting research has tracked wellbeing across the adult lifespan, often analysing data from tens of thousands of participants from late adolescence (approx. 16–18 years) into old age [7–11]. Trajectories of wellbeing that occur during adolescence itself (10–24 years, [12]) have, however, been routinely given less focus in the research process, even though this time of life harbours some of the most wide-reaching changes in brain structure and function [13], cognitive abilities [14,15], sociality [16] and mental health [17,18]. As these changes can have long-lasting influence, an understanding of how subjective wellbeing develops in adolescence is important for supporting wellbeing across the whole life course [19,20]. We, therefore, present analyses that provide a new view of wellbeing developments during this critical period of life.

### 1.1. Defining life satisfaction

A central question for social scientists and policymakers alike concerns the extent to which policies, interventions and research efforts succeed in improving people's lives. To answer these questions, improvements must be quantified, yet no single domain captures the quality of a person's life in its entirety. Indeed, it is reasonable to assume that specific policies and interventions may affect different desirable life outcomes—outcomes such as good health, stable financial resources or strong social relationships—in different ways. For this reason, many social scientists have considered whether it is possible to develop broad measures that capture the overall levels of wellbeing that people experience.

There are many ways to conceptualize and operationalize wellbeing (for an overview, see [5]). For instance, affective approaches focus on the emotions that people experience on a moment-to-moment basis [21]. Wellbeing, then, could be defined simply as experiencing a preponderance of pleasant feelings over unpleasant feelings [22]. Objective-list-based approaches, on the other hand, require that experts identify which components of life are necessary for one to be ‘well,’ and then those components are assessed directly. Those with high wellbeing, according to this approach, are those who acquire these components, including things such as good health, adequate income, supportive social relationships and freedom and autonomy.

An alternative to these approaches prioritizes respondents' own subjective evaluation of whether their lives are going well. These subjective approaches allow respondents themselves to determine which domains in life are most important and how well they are doing in those domains [6,23]. One increasingly common version of this subjective approach is to assess self-reported judgements of life satisfaction. For instance, life satisfaction can be assessed quickly and simply by asking ‘how dissatisfied or satisfied you are with … your life overall’. A broad range of research on the correlates of these measures supports their construct validity as indicators of overall wellbeing [6].

### 1.2. Life satisfaction across the lifespan

Because the circumstances that are thought to predict wellbeing change systematically with age, much research has tracked life satisfaction across the adult lifespan [7–11]. These studies have identified somewhat of a paradox regarding the patterns of changes that emerge. Specifically, many (though not all) studies have found a U-shaped curve in life satisfaction ratings, whereby life satisfaction declines from adolescence through early and middle adulthood, followed by a rebound in older adulthood [9,24–26]. The pattern is seen as somewhat of a paradox because many objective circumstances (including income) increase during the period of life when life satisfaction exhibits its greatest decline, whereas other circumstances (including health status) decline when life satisfaction is rebounding in late life. These patterns have been observed in samples from many different countries ([7,9], yet see [27–29]). Although methodological concerns—including concerns about the impact of cohort, period and instrumentation effects—have been raised, the robustness of this pattern across studies that rely on different populations and analytic approaches suggests that at the very least, it cannot be explained entirely by methodological artefacts.

### 1.3. Adolescent life satisfaction

Work tracking life satisfaction from when participants enter large-scale panel surveys (often in late adolescence at 16 or 18 years), has, therefore, highlighted that life satisfaction drops in late adolescence and early adulthood: a decline that continues into middle age [9,24–26]. This decrease in life satisfaction can be explained (at least partially) by mechanisms including growing social, financial, professional or family pressures and changing appraisals of life satisfaction questions [30]. Social capital, such as peer relationships, may be one of the strongest predictors of life satisfaction in adolescence [31]. Furthermore, adolescence is a time of social re-orientation [16]. Temporary dissatisfaction with social relationships, may motivate adolescents to seek new social roles and build lifelong relationships [32].

However, there has yet to be a comprehensive study to map how life satisfaction develops in early adolescence, and how this compares to the more widely studied developments across the lifespan. A handful of large-scale longitudinal studies have studied earlier age ranges (e.g. 15–18 years; [8,33–36]), or examined adolescents specifically [36,37]. The resulting evidence suggests that the decline in subjective wellbeing that has been found among young adults (e.g. those over 15 years), may actually begin even earlier and subsequently continue throughout adolescence [33–36]. However, when exactly large drops in life satisfaction occur remains unclear as most studies have yet to examine the whole adolescent age range of 10–24 years [12] and adolescents' transition into adulthood [38].

Further, differences in wellbeing trajectories between boys and girls have been routinely noted [39]. Multiple studies suggest that adolescent girls do significantly worse than adolescent boys in terms of subjective wellbeing and internalizing mental health [33,34,40–43]. This has been supported in recent work with data from 73 countries [40]. Campbell and colleagues also, and perhaps counterintuitively, show that higher country-level gender parity and GDP are associated with a larger difference in wellbeing between boys and girls. Whether this finding is an artefact of the, largely, cross-sectional modelling, or rather owing to intrinsic mechanisms such as disparities in expectations and reality in high-party–high-GDP countries [44,45], is unclear. A better understanding of how life satisfaction develops at the individual level, whether and how these trajectories differ between boys and girls and if these differences persist into adulthood, is, therefore, needed [10].

### 1.4. The current study

To establish a deeper understanding of life satisfaction trajectories in adolescence, the current study introduces three core innovations to the literature: (i) it analyses data collected from 37 076 adolescent participants aged 10–24 years from the UK and Germany, to understand adolescent life satisfaction trajectories, and compares these with trajectories found in adulthood (95 466 adult participants aged 25 or over); (ii) it uses longitudinal modelling to differentiate within- and between-person patterns and understand in-person change over the whole period of adolescence; and (iii) it investigates the sex differences highlighted in previous adolescent mental health research to understand possible disparities. The UK dataset was analysed in an exploratory fashion; the analyses were then pre-registered to replicate on a second German dataset. In doing so, we rigorously test whether adolescence harbours large-scale changes in life satisfaction trajectories and if sex differences are evident during this process.

## 2. Methods

### 2.1. Data

This study analysed data from the UK Understanding Society longitudinal household panel survey [46] and the German Socio-Economic Panel (SOEP) study (International Science Use Version; [47]). See table 1 for a comparison of the two. The Understanding Society survey is an annual longitudinal study of about 40 000 UK households started in 2009 [46]. The data are collected annually, however, because of its large scale each wave is collected over 2 years. This means that the collection of one wave (e.g. the 2009 wave) extends into the next year (e.g. 2010) at which point the next wave (e.g. the 2010 wave) has started data collection. We use nine waves (2009–2018) of data as released in February 2020. The study consists of an adult questionnaire completed by anyone 16 and over in the households surveyed and also a youth survey that is filled out by 10–15-year-old adolescents in each household. Participants can age in and out of certain surveys, e.g. completing the youth survey and then graduating into the adult survey. At study onset, the population sampled was recruited to ensure accurate representation of the UK population. It now consists of multiple different samples: the General Population Sample, the Ethnic Minority Boost Sample, the former British Household Panel Survey sample and an Immigrant and Ethnic Minority Boost Sample (for more information see [48]).

**Table 1.** Comparison of Understanding Society and German Socio-Economic Panel datasets.

	Understanding Society	German Socio-Economic Panel (SOEP)
country	United Kingdom	Germany
number of households surveyed	∼40 000	∼15 000
number of participants analysed	91 267	41 275
number of adolescent participants analysed (10–24 years)	27 130	9946
number of adolescent participants analysed (10–21 years)	22 531	7974
number of adolescent participants with one wave of data analysed (10–21 years)	6720	5036
number of adolescent participants with two waves of data analysed (10–21 years)	4210	2011
number of adolescent participants with three+ waves of data analysed (10–21 years)	11 601	927
first year of data analysed	2009	2016
number of waves analysed	9	3
distance between waves	annual (but each wave is collected over 2 years)	annual
ages available	10+ years	12, 14 and 17+ years
life satisfaction scale	7-point scale	10-point scale

Similar to the UK data, the German data are also a household panel survey. It was started in 1984 and now surveys about 30 000 people and 15 000 households annually. The sample has been refreshed multiple times to ensure an accurate representation of the German population. In the German data, participants under 18 were questioned if they had turned 12 years (pre-teen questionnaire), 14 years (early youth questionnaire) or 17 years (youth questionnaire) in each survey year, after which participants graduated into an annual adult questionnaire. There is, therefore, no data for ages 13, 15 and 16. As data collection from 14-year-olds only commenced in 2016, we analysed three waves of data (2016–2018, as released in December 2020; we mistakenly pre-registered that we will use data from 2015 onwards owing to ambiguity in consulted data documentation). Both studies have been used frequently to examine age differences in life satisfaction among adults (e.g. [8,30,49–52]).

### 2.2. Measurement

#### 2.2.1. United Kingdom (Understanding Society)

We use life satisfaction, age and sex measures from both the youth and adult survey of the UK Understanding Society study. We chose our life satisfaction question because it was harmonized across ages and the two datasets (Understanding Society and SOEP). In the UK youth survey, adolescents used a 1–7 visual scale (very happy smiley face—very sad smiley face; scale subsequently reversed) to answer ‘which best describes how you feel about your life as a whole?’; in the adult survey participants were asked ‘how dissatisfied or satisfied you are with the following aspects of your current situation … your life overall’ on a 1–7 numeric scale. We treated the life satisfaction scores provided in the youth and adult survey as continuous, owing to previous simulation work showing that variables with more than five categories can be approximately represented by continuous analytic methods [53]. We assumed the adult and youth questions were not substantially different, and the electronic supplementary material provides tests to support this assumption and also further information about the measures.

The study includes two variables to address the selective dropout of certain populations during data collection, by including them as auxiliary variables (explained below). Firstly, it includes the log of monthly household income provided by an adult survey respondent (those with zero monthly income were assigned the next lowest reported income level to allow for log transformation). Secondly, it also includes the Index of Multiple Deprivation for the participant's residential area. To derive this value, we extracted the participants' lower super output area (LSOA) from the Special License version of the Understanding Society survey and merged it with data provided by authorities in England, Scotland, Northern Ireland and Wales specifying the area's general Index of Multiple Deprivation.

Age was derived by Understanding Society from self-reported date of birth for the youth and adult sample. Further sex was taken from the ‘stable characteristics' file provided by Understanding Society which integrates all waves of data collection to ensure the most accurate report is used. In the youth survey, participants reported annually whether they were ‘male’ or ‘female’; in the adult sample they were asked to confirm their sex collected previously or report it as ‘male’ or ‘female’. They were allowed to refuse to respond. If sex reporting was inconsistent, i.e. if it varied between waves, the sex was labelled as ‘0’ in the stable file and we recode these instances as NA (30 measurement occasions).

#### 2.2.2. Germany (SOEP)

Each of the German questionnaires included a largely identical question about life satisfaction at the end of the data collection process. For example, in the pre-teen and early youth questionnaire it was introduced as: ‘In conclusion, we would like to ask you how satisfied you are with your life in general. Please give your answer on the basis of the following scale. The value 0 means ‘completely dissatisfied’. The value 10 means ‘completely satisfied’. By means of the values between 0 and 10 you can rate your answer between the two extremes. How satisfied are you with your life?’ (0–10 scale; see the electronic supplementary material). A monthly household income variable, retrieved from the generated household data file provided annually by the SOEP study, was used to address selective dropout (by including these as auxiliary variables as explained below; the Index of Multiple Deprivation was not available in Germany).

In the data, age was derived differently for those participants in the adult versus pre-teen, early youth and youth questionnaires. For the adult participants, age was calculated by subtracting the year the questionnaire was completed by the year of birth collected in the autobiographical data file for each individual. For the younger participants, we assigned those ages they would turn in the survey year in question, i.e. 12 years if they completed the pre-teen questionnaire, 14 years if they completed the early youth questionnaire and 17 years if they completed the youth questionnaire. This approach ensured we had the necessary number of participants, and data structure appropriate for our longitudinal modelling later in the analysis pipeline. Sex was provided in the autobiographical dataset present for each participant.

### 2.3. Inclusion criteria

Before proceeding to analyse the data, we applied a variety of inclusion criteria. In the UK dataset, we only included those participants that were in the age range specified for the survey they filled out: i.e. we included from the youth survey only participants over the age of 9 years and under the age of 16 (excluded 61 measurement occasions) and only included those from the adult sample that were over the age of 15 (excluded 65 measurement occasions). For those UK participants who completed the survey twice within the same 1-year age bin, we excluded the second time the data were collected (excluding 417 989 measurement occasions). This was necessary as our longitudinal modelling technique only allowed one measurement occasion per participant per age. We did not need to apply either exclusion criteria to the German data as there was no participant who fit these requirements.

In both datasets, we excluded participants over 80 to generally match UK life expectancy (UK: excluding 15 537 measurement occasions; Germany: excluding 2596 measurement occasions), to ensure there were enough participants per age group. Lastly, in the UK data, we deleted all those cases where sex was NA (31 measurement occasions) because these participants could not be assigned to a specific sex during the analyses. Sex could be NA because, for example, a participant changed their reporting on the sex (male or female) item during the study or because they decided to refuse response to this item. There was no participant who had missing data for sex in the German dataset.

After exclusions, in both the datasets, we are left with 417 989 measurement occasions from a total of 91 267 UK participants aged 10–80, and 89 869 measurement occasions from a total of 41 275 German participants aged 12–80. Not all participants provided data for the same number of waves. In the UK dataset, we had the following number of participants per wave: one wave = 19 566; two waves = 12 220; three waves = 8927; four waves = 7692; five waves = 6241; six waves = 6130; seven waves = 7369; eight waves = 11 232; nine waves = 11 890. In the German dataset, there were the following numbers of participants per wave: one wave = 11 622; two waves = 10 712; three waves = 18 941.

### 2.4. Longitudinal analyses

To analyse the longitudinal adolescent data (UK (Understanding Society): age 10–24 years, Germany (SOEP): age 12–24 years) we fitted a series of latent growth curve models [54,55] using the R package *lavaan* [56]. We estimated our model using robust full information maximum likelihood (FIML) with robust Huber–White standard errors to account for deviations from multivariate normality. Further, we used auxiliary variables (UK (Understanding Society): log of total household net income and Index of Multiple Deprivation, Germany (SOEP): log of total household net income) as part of the FIML missing data imputation to account for selective dropout of certain populations owing to socio-economic status and income in the longitudinal sample [57]. The use of auxiliary variables (i.e. variables that are not included in the model but are added to allow FIML to take them into account when imputing missing data) has been shown to outperform procedures such as the application of attrition weights [58]. Owing to the nature of our modelling approach, which modelled across age rather than time, we were not able to integrate sampling weights into our estimation strategy. This limits the extent to which we can generalize our findings to national populations. We also note here that some adolescents in the datasets are from the same household (see the electronic supplementary material).

To assess model fit we report the chi-square test with degrees of freedom, root mean square error of approximation (RMSEA) and its confidence interval, the Comparative Fit Index (CFI) and the standardized root mean squared residuals (SRMR). We report model fit [59] as follows: RMSEA < 0.05 (acceptable: 0.05–0.08), CFI > 0.97 (acceptable: 0.95–0.97) and SRMR < 0.05 (acceptable: 0.05–0.10), although we note that latent growth model may show poorer fit in some circumstances [60]. Our growth modelling approach allowed us to capture change over time. As we hypothesized change would probably be nonlinear, we fitted a series of different models. This included a standard linear growth model (with slope loadings 0, 1, 2, etc.), a quadratic growth factor added to the linear factor, as well as a latent-basis model. The latter model is the most flexible and a suitable model if linear or polynomial change may be too restrictive (e.g. [61]): by only constraining (usually) the first and last factor loadings, it allows us to capture a wide range of nonlinear shapes (e.g. cf. [54, p. 594]; [61, pp. 11–12]) with the ‘price’ of added complexity in terms of a greater number of parameters. We compared a series of models that vary in both the complexity of the functional form, as well as in the restrictiveness of other model properties (e.g. equality constraining of error variances across time and/or groups), and use model selection to decide on the optimal balance of model fit versus complexity (e.g. see [61,62]).

### 2.5. Sex differences

To examine when sex differences emerge, we fitted two types of model to data at each age. One model predicted life satisfaction by taking into account sex (*life satisfaction ∼ 1* *+* *sex*) while the other model did not include this factor (*life satisfaction ∼ 1* *+* *0* * *sex*, i.e. only computing the intercept and not regressing sex onto life satisfaction). We use Akaike information criterion weights (AIC weights, [63]) to test whether a version of such a model that includes sex outperforms a model that does not include sex. We also use the ratios of these AIC weights to show by how much the model with sex differences incorporated is favoured. If there is no substantial sex difference in a given parameter, this procedure will favour the more parsimonious model that assumes no such differences; if there is a sex difference, then the greater explanatory power will outweigh the added complexity of additional parameters [62]. Within this framework, values below one favour a model without sex, values above one favour a model that includes sex [63].

We continued by running longitudinal latent growth curve to examine the sex differences highlighted. As these were more exploratory for the UK data, we first fitted models to 20% of data before expanding to the whole dataset, at which time we added some minor changes to the model owing to fit. We chose 20% as this balanced the need for adequate statistical power in the model-building dataset, against the aim to subsequently test the models on a larger dataset.

We first examined the functional form (linear, quadratic or latent-basis model), comparing the models using a likelihood ratio test. We progressed using a latent-basis model and then freed or constrained error variances between waves and groups. Lastly, we used a similar approach to compare group differences in substantive parameters (e.g. mean/variance of the slope or intercept parameters). In the German data, we followed a pre-registered approach, first examining functional form (linear, quadratic or latent-basis model) using likelihood ratio tests. We then tested freed versus constrained error variances between waves and groups, progressing to testing whether sex differences are present by freeing and constraining slope loadings across sex. As a model that had the same slope loadings for both sexes was found to be preferred, we did not proceed with fitting further model iterations.

### 2.6. Code and pre-registration availability statement

The data cleaning and analysis code are available on the OSF: https://osf.io/9kd2r/. Furthermore, this manuscript is fully reproducible and the appropriate R markdown is available on the OSF. The pre-registration of the analyses to be performed on the German data is also available on the OSF: 10.17605/OSF.IO/RN29V. See the electronic supplementary material for the Data accessibility section.

## 3. Results

### 3.1. Cross-sectional life satisfaction levels

**Figure 1.**
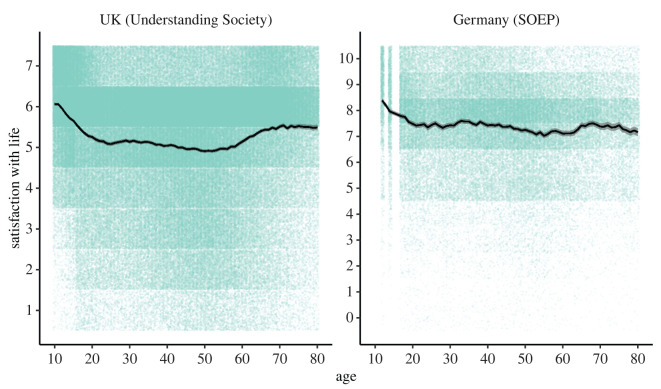
Cross-sectional trajectory of life satisfaction for both UK (Understanding Society) and German (SOEP) participants between the ages of 10 and 80 years, and the ages of 12 and 80 years, respectively (black line = mean value by age, grey ribbon = 95% confidence interval error bars, teal points = individual data points). Please note that the error bars are very small owing to the large sample size analysed.

While most previous studies of lifespan life satisfaction have examined adult populations, with a minority investigating the ages 15 or 16 onwards, figure 1 shows that the difference in life satisfaction scores between the beginning and end of adolescence is substantial (UK (Understanding Society): life satisfaction at age 10 = 6.07 versus at age 24 = 5.09; difference equates to 0.84 s.d. using the s.d. at age 10; Germany (SOEP): life satisfaction at age 12 = 8.39 versus at age 24 = 7.35; difference equates to 0.63 s.d. using the s.d. at age 12). Indeed, there is no other time between adolescence and adulthood where differences for similarly short intervals are so pronounced in either dataset. The electronic supplementary material provides additional analyses to address concerns about potential repeated measures, instrumentation effects and clustering by household (electronic supplementary material, figures S1–S3, respectively). In the electronic supplementary material figure S4, we show a random selection of 75 UK adolescent participants' raw life satisfaction scores, highlighting large variation in scores on an annual basis.

### 3.2. Longitudinal life satisfaction trajectories

While cross-sectional analyses allow us to examine associations between age and life satisfaction, longitudinal analyses are necessary to track true within-subject trajectories, which need not match cross-sectional associations unless strict assumptions (e.g. ergodicity) are met [64,65]. Throughout the study we analysed the UK data first, developing an analytical strategy that was then pre-registered and applied to the German dataset. For both the UK and the German data, the best fitting model determined by a likelihood ratio test was a latent-basis model, where only the first (0) and last (1) loadings were constrained and all others were freely estimated. In the UK data, we fitted a model with freed error variances and in the German data we fitted a model with constrained error variances to allow for better model convergence. The fit measures were good, except the SRMR measure for the German dataset, which could be a result of large residuals owing to the 2 years of missing data between the ages of 14 and 17 (UK (Understanding Society): $\chi_{102}^{2}=1061.358,$ *p* < 0.001, RMSEA = 0.019 [0.02, 0.018], CFI = 0.91, SRMR = 0.069; Germany (SOEP): $\chi_{51}^{2}=102.004,$ *p* < 0.001, RMSEA = 0.01 [0.013, 0.007], CFI = 0.961, SRMR = 0.199).

**Figure 2.**
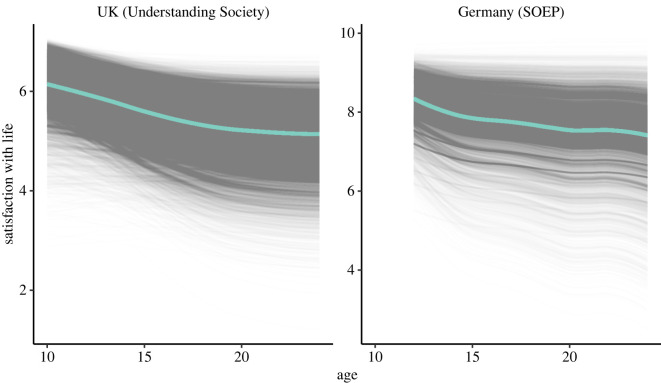
Longitudinal life satisfaction trajectories in UK and German data, for the ages of 10–24 years and 12–24 years, respectively. Latent growth curve estimated life satisfaction trajectories for adolescents (black lines) and trajectory for a simulated adolescent with a mean life satisfaction at ages 10 and 12, respectively (teal line).

The UK dataset baseline model estimated a life satisfaction score of 6.09 (s.d. = 0.75) at age 10, corresponding to an average response between 6 (smiling face) and 7 (very smiling face) on a 7-point visual scale. Yet, there are pronounced between-person differences in life satisfaction at this time point (variance = 0.56, s.e. = 0.02, *p* < 0.001). To study how this change unfolds over time, we fitted a latent-basis model which allowed us to estimate a flexible, non-linear shape of change, which is shown in figure 2 (left). The model shows a near universal decrease of life satisfaction between the ages of 10–24. The most prominent decrease in life satisfaction occurs early in adolescence while the decrease decelerates in later adolescence (see the electronic supplementary material, table S1 for slope loadings). The mean slope (est. = −0.98, s.e. = 0.03, *p* < 0.001) as well as the slope variance (est. = 0.97, s.e. = 0.07, *p* < 0.001) is pronounced, suggesting both population level change and considerable individual differences in the degree to which individuals expressed this change. We observed a substantial negative correlation between the adolescents' starting point at age 10 and their trajectory (est. = −0.31, s.e. = 0.03, *p* < 0.001): those adolescents with a higher initial life satisfaction show a steeper decrease throughout adolescence. To visualize these individual differences in life satisfaction patterns, we plot the model implied trajectories for each individual (figure 2, left, black lines) as well as the trajectory for a simulated participant starting at the population mean and population mean slope (figure 2, left, teal line).

The model fitted to German data estimated a life satisfaction score of 8.36 (s.d. = 0.97) at age 12 (between 8 and 9 on a 10-point numerical scale from 0 = ‘completely dissatisfied’ to 10 = ‘completely satisfied’). Many of the characteristics of the model estimates were similar to the UK data (figure 2, right). There were prominent between-person differences in life satisfaction at age 12 (variance est = 0.93, s.e. = 0.16, *p* < 0.001) and life satisfaction decreased more quickly in early adolescence compared to late adolescence (see the electronic supplementary material, table S2 for slope loadings, mean slope: est. = −0.97, s.e. = 0.07, *p* < 0.001). However, the variance in slope (est. = 1.02, s.e. = 0.58, *p* = 0.078) and the correlation between an adolescent's starting point at age 12 and their trajectory were non-significant (est. = −0.06, s.e. = 0.28, *p* = 0.822).

### 3.3. Sex differences

**Figure 3.**
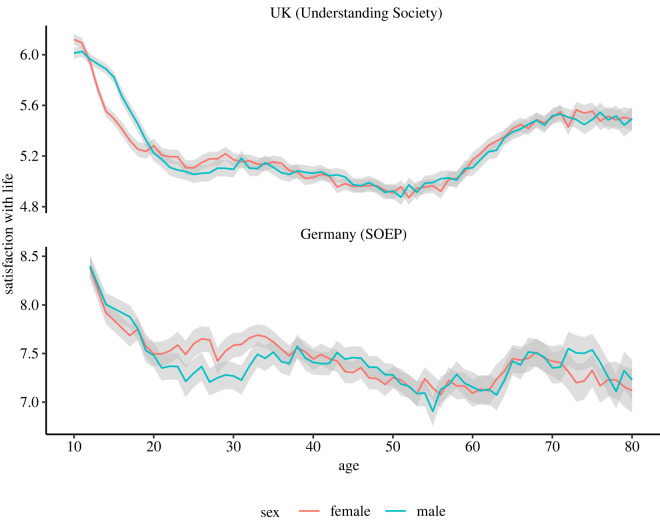
Life satisfaction scores by age for 91 267 10–80 year-olds UK, and 41 275 12–80 year-olds German participants. Top: the UK data split by sex show that adolescence is also a time of substantial sex differences with girls showing an earlier drop in life satisfaction scores than boys, which lasts until late adolescence when the sex differences get smaller, and males do slightly worse than females in terms of life satisfaction. Bottom: the German data present less clear sex differences in early adolescence than the UK data, even though girls do show lower life satisfaction than boys during that time. The German data, however, show a prominent sex difference in life satisfaction during late adolescence and early adulthood, when males score lower in life satisfaction than females.

Having investigated the trajectories of life satisfaction in adolescence, one relevant avenue for further investigation is the sex differences that have been highlighted in previous work [39]. When comparing for sex in the cross-sectional UK data, we find a prominent difference emerging in early adolescence: girls show a more pronounced drop in life satisfaction scores than boys, but this difference levels off in later adolescence when boys start reporting lower life satisfaction than girls at times (figure 3, top). In the German data, the decrease in life satisfaction for young adolescent girls is less distinct from boys (figure 3, bottom): this could be owing to an absence of effect or the lower density of data collected (adolescent data in the German sample was not collected annually, and was only available for those aged 12, 14 and 17). The German data, however, have clearer evidence of older adolescent and young adult males scoring lower in life satisfaction than their female counterparts, complementing the trend found in the UK data. Additional analyses in the electronic supplementary material shows that alternative explanations such as repeated measurements (electronic supplementary material, figure S5), instrumentation effects (electronic supplementary material, figure S6) and household clustering (electronic supplementary material, figure S7) do not affect this finding. Further, while adolescent sex differences in life satisfaction appear (and disappear) at slightly different age groups for different UK birth cohorts, they are consistently present throughout the data collected (electronic supplementary material, figure S8).

Applying an AIC weights approach to UK data we find that there is an overwhelmingly strong preference for a model allowing for sex differences to exist between the ages of 13 and 18 (AIC weights ratios between 39 000 and 3 trillion, see also the electronic supplementary material, table S3 and figure S10). While some other ages like 10 (ratio = 139.97), 27 (ratio = 7.85) or 29 (ratio = 15.12) additionally show modest evidence for sex differences, the evidence for sex differences in the UK data is clearly most compelling in the mid-adolescence range from 13 to 18 years. In the German data, the most compelling sex differences were found in adulthood: at ages 25–27 (ratio at 25 = 25.72, ratio at 26 = 19.99, ratio at 27 = 5,186.66) and 30–32 (ratio at 30 = 86.41, ratio at 31 = 446.95, ratio at 32 = 52.44). In the mid-adolescent age range, there was little evidence for sex differences being apparent in the German data (ratio at 12 = 0.38, ratio at 14 = 0.63, ratio at 17 = 5.14; see also the electronic supplementary material, table S4 and figure S11).

#### 3.3.1. United Kingdom (Understanding Society)

To understand the sex differences in life satisfaction trajectories in the UK data in more detail we harnessed the longitudinal scope of the data by fitting multi-group latent growth curve models to participants between the ages of 10 and 21 years for females and males separately (13–18 years ± 3 years). We built our models on 20% of the data to test the modelling approach, before fitting and adjusting them on the whole data (we note here that fitting models on 80% of the data yielded virtually identical results). The total sample for this analysis was 72 476 measurement occasions from 22 531 adolescents. Our final model's fit statistics were acceptable ($\chi_{127}^{2}=810.55,$ *p* < 0.001, RMSEA = 0.02 [0.02, 0.02], CFI = 0.92, SRMR = 0.06).

To compare group differences and similarities we selectively freed, and constrained, parts of the latent growth curve model within (over time) and between groups using a likelihood ratio test. First, we examined life satisfaction at baseline by freeing the mean of the intercept. Doing so, we find that females have a higher life satisfaction than males at age 10 ($\Delta \chi_{1}^{2}=24.36,$ *p* < 0.001; female: est. = 6.16, s.e. = 0.02, *p* < 0.001; male: est. = 6.02, s.e. = 0.03, *p* < 0.001, the group difference was moderate in size: 0.19 pooled s.d.). However, the variance of the mean life satisfaction at age 10 did not differ between sexes ($\Delta \chi_{1}^{2}=0.03,$ *p* = 0.85; est. = 0.53, s.e. = 0.02). Second, we examine slope loadings. We find that a free basis model with different patterns of loadings between sexes fits better ($\Delta \chi_{10}^{2}=241.02,$ *p* < 0.001) than one that assumes the same developmental shape (for a comparison of slope loadings see the electronic supplementary material, table S5). Although direct comparisons of sex differences in slope intercept and variance are not straightforward given the specific model constraints with regard to slope loadings, we observe substantially more negative slopes in females than males, meaning that they decrease at a faster rate earlier in adolescence ($\Delta \chi_{1}^{2}=10.36,$ *p* = 0.001; female: est .= −0.99, s.e. = 0.04, *p* < 0.001; male: est. = −0.82, s.e. = 0.04, *p* < 0.001). Moreover, males showed greater variance for their slope estimates than females; they, therefore, show more within group differences in their life satisfaction trajectory over the time span examined (constrained model failed to converge; female: est. = 0.80, s.e. = 0.09, *p* < 0.001; male: est. = 1.15, s.e. = 0.12, *p* < 0.001). Lastly, we find that these sex differences remain almost identical when we use a model with equality constrained slope loadings (electronic supplementary material, figure S9).

**Figure 4.**
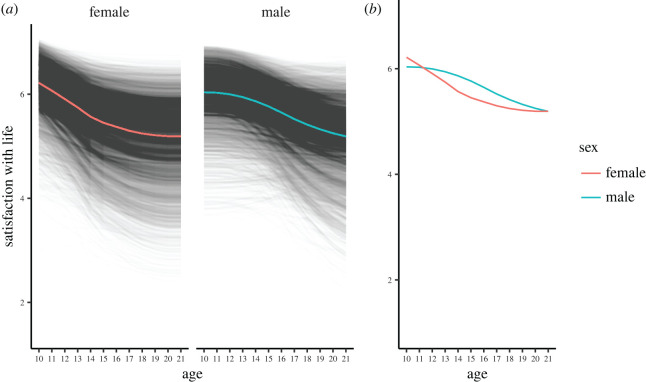
Longitudinal latent growth curve models of life satisfaction between 10 and 21 years in the UK dataset, differentiating by sex. (*a*) Latent growth curve model implied trajectories of life satisfaction for all participants (split by sex). The coloured lines show the implied trajectory for a simulated adolescent girl or boy who starts at their sex's mean life satisfaction at age 10. (*b*) The model implied trajectories for a simulated adolescent girl and boy who starts at their sex's mean life satisfaction at age 10 are overlaid to accommodate comparison.

The differences between the life satisfaction trajectories in males and females can be seen when plotting the model implied trajectories of the participants in the data. Females show a quicker decrease from a higher initial life satisfaction, while males show a later decrease but more variance in the amount they decrease over adolescence (figure 4). The coloured lines in figure 4 that plot the trajectory for a simulated adolescent starting at their sex's mean and sex's mean slope reinforce this trend and show that female and male differences decrease in late adolescence and ultimately disappear around the age of 21 (figure 4*a* coloured lines, and figure 4*b* colour lines overlaid to aid comparison). Taken together, our findings from UK data would suggest that during adolescence males and females differ in the nature of their life satisfaction trajectory, the extent to which they express these changes and the degree to which they differ from each other.

#### 3.3.2. Germany (SOEP)

We pre-registered the approach taken above on the UK data, to be repeated on the German data to examine whether sex differences would also be present. We fitted free basis latent growth curve models with constrained error variances that either constrained the slope loadings to be equal across sex, or allowed the slope loadings to vary. We find that the free basis model with the same patterns of slope loadings between sexes does not fit significantly worse than one that assumes different developmental shapes ($\Delta \chi_{5}^{2}=6.39,$ *p* = 0.270). We, therefore, invoked our pre-registered plan not to continue further model testing procedures, as there was no clear evidence for sex differences. As stated above, this lack of sex differences could be owing to a lack of differences, cultural differences in when and how sex differences manifest, or because the dataset was both smaller (22 531 UK participants aged 10–21 years versus 7974 German participants aged 12–21 years) and has a lower density of data collected (mean number of waves per participant: 3.22 UK (Understanding Society) versus 1.48 Germany (SOEP)), especially for the younger adolescent age range of interest where the German dataset had complete missing data for ages 13, 15 and 16 years.

## 4. Discussion

This study analysed two large-scale longitudinal datasets with up to nine waves of annual life satisfaction data from 37 076 UK and German adolescent participants aged 10–24 years and 95 466 adult participants aged 25 years or over. Cross-sectionally we find that adolescence harbours the steepest and most pronounced drops in life satisfaction across the whole adult lifespan in both datasets, and that this drop is steepest before the time frame normally studied by longitudinal surveys (16–18 years or older). Only in the UK dataset do adolescents also show substantial sex differences in life satisfaction levels: girls start at a slightly higher baseline life satisfaction at age 10, but show an earlier decrease in life satisfaction than adolescent boys. In both datasets, male life satisfaction trajectories ‘catch up’ later in adolescence when life satisfaction scores converge across sexes, and in the German dataset female life satisfaction starts outperforming male life satisfaction when moving into adulthood.

Some of the differences between datasets, especially with regards to the sex differences in trajectories of life satisfaction, could be owing to the diverging dataset characteristics (table 1). Specifically, the German dataset was substantially smaller (7974 participants aged 10–21 years, with 927 of those having three waves of data available), meaning it had lower statistical power to detect small effect sizes. Further, the German dataset was missing data for certain ages (13, 15 and 16 years), meaning it had fewer waves per person, and could, therefore, be less suitable for detecting differences in trajectories during this period.

Model differences between our UK and German sample, such as the correlation between model intercept and slope that was only significant in the UK sample, could be owing to additional dataset differences. For example, the UK measure of life satisfaction had a smaller range of response options (7-point scale) than the German dataset (10-point scale), which could have led to a ceiling effect. This explanation, however, seems unlikely to explain the entirety of the difference as only few adolescent participants scored the maximum life satisfaction score (27.9% of 10–21 year-olds in the UK dataset; 15.3% in the German dataset) and recent evidence suggests that the benefits of having more than six response options on a Likert scale are negligible [66]. It is more likely that dataset characteristics highlighted above were influential in the resulting model differences. For example, the fewer number of waves per person in the German dataset could have led to slopes not being estimated with high reliability in that sample, resulting in the low correlations.

### 4.1. Life satisfaction decreases in adolescence

There are two different explanations for why adolescents show drastic decreases in life satisfaction throughout the adolescent age range. First, the drop in life satisfaction scores during adolescence could be driven by conditions of life getting worse during this period, e.g. increasing social insecurity, autonomy or uncertainty, or the change being a consequence of certain developmental changes [67,68]. While life satisfaction is not identical to mental health, adolescents experience prominent increases in mental disorders such as depression or anxiety and decreases in other forms of subjective wellbeing [41,42,69–71]. This supports the notion that life satisfaction drops owing to decreases in quality of life. We also find that decreases in life satisfaction are steepest in early adolescence, which presents a period of social re-orientation; the decrease in life satisfaction may be symptomatic of a motivation to engage in developmental tasks such as individuation, exploration and connection to peers [32].

Second, the appraisal process that determines the response to a life satisfaction question might also change throughout adolescence owing to alterations in cognitive or social processes [15,72,73]. For example, the continued development of the ‘social brain’ in adolescence allows for increased and improved mentalizing, i.e. adolescents' skill in understanding how others think and feel [16,74,75]. Further, adolescents’ interest in peers increases and so does the influence of their peer group on behaviour [33,76,77]. A decrease in life satisfaction scores could, therefore, also be linked to changes in cognitive and social appraisals of what a question about one's satisfaction with life means, such as an increased sense of a larger social world or comparing one's own life to more stringent and competitive benchmarks. Both of these explanations could not be tested in the current dataset but would merit future investigation.

### 4.2. Sex differences in life satisfaction trajectories

Our study adds another perspective to studies highlighting a difference between boys' and girls’ wellbeing in adolescence, where girls score systematically lower on subjective wellbeing questionnaires than boys [40]. Our cross-sectional UK adolescent data support this finding: yet longitudinally it becomes apparent that the gap closes later in adolescence and might even reverse in young adulthood (epidemiological studies have found such a narrowing of the difference between sexes when age increases [78]). The German data provide less evidence of a difference between sexes in early adolescence; it, however, provides evidence for the reversal in early adulthood when young men report lower life satisfaction than women.

Overall, these findings highlight that cross-sectional analyses may overestimate sex differences (cf. [40]). The UK data, in particular, indicate that decreases in life satisfaction in boys simply occur later than they do in girls. This might be symptomatic of ‘accelerated maturation’ of adolescent girls. As girls proceed through certain developmental stages, e.g. puberty [79], earlier than their male counterparts, this might induce an earlier decrease in life satisfaction. While the understanding that there is a gap between boys' and girls’ wellbeing encourages researchers and policymakers to focus on boosting female life satisfaction and wellbeing throughout adolescence, an ‘accelerated maturation’ hypothesis emphasizes that male decreases in life satisfaction should not be discounted. Males, for example, have a higher incidence of externalizing problems (e.g. alcohol and substance use) than females [17,18,78], and these difficulties can emerge later in adolescence. Interventions might thus need to be adapted in both scope and timing to fit their needs.

### 4.3. Limitations

This study, therefore, opens many avenues for potential research, including examining how major changes in cognitive and social appraisal during development might be altering adolescents' response to life satisfaction questions. There is also potential for future research to address some of the limitations of this study. Firstly, our work relies on a single item measure of life satisfaction; while this measure triumphs in its ease of data collection, a more diverse set of measurements would be helpful to gauge measurement changes, challenges and nuances. For example, the interpretation and nature of life satisfaction might be changing across the lifespan, something that could be examined using measurement invariance with a more extensive measure. Further, owing to harmonization, we could only focus on life satisfaction, however, mental health or other wellbeing measures would have been insightful to include. Finally, the data were collected from UK and German populations, but to guide a global understanding of life satisfaction a greater variety of countries would need to be studied.

## 5. Conclusion

The last decade has seen adolescence become progressively recognized as a core developmental stage; adolescence is a period where many of the key health, social and developmental trajectories experienced in adulthood first crystallize [80–82]. This recognition has led to large research and funding investments that aim to improve adolescents’ mental health, cognitive and social outcomes [83,84]. In parallel, there has been interest in measuring and improving global wellbeing levels, with initiatives focusing primarily on adults (e.g. [2]). These two developments have been co-occurring without much reciprocal recognition, as many of the large-scale longitudinal studies of wellbeing trajectories only analysed those participants aged over 16 or 18 years. This study, however, shows that early adolescence harbours important and substantial changes in subjective wellbeing that need to be further understood. To start mapping and comprehending wellbeing across the lifespan, and with it the near-universal downward trajectories of adolescent life satisfaction highlighted in this paper, behavioural scientists need to recognize the urgent need to start merging adult and adolescent research perspectives.

## Data Availability

Understanding Society data are distributed by the UK Data Service. Most of the data are available for download, after the completion of a registration form, from the UK Data Service: the University of Essex, Institute for Social and Economic Research, NatCen Social Research, Kantar Public. (2019). Understanding Society: waves 1–9, 2009–2018 and harmonized BHPS: waves 1–18, 1991–2009. (data collection). 12th edition. UK Data Service. SN: 6614, http://doi.org/10.5255/UKDA-SN-6614-13. The Special License data necessary to calculate the Index of Multiple Deprivation are available after a more intensive approval procedure. The SOEP data are available for download, after the completion of a registration form and data distribution contract from DIW Berlin (International Science Use Version; Goebel, Jan *et al.* [85]). Electronic supplementary material is available online at [86].
